# TLRs as a Promise Target Along With Immune Checkpoint Against Gastric Cancer

**DOI:** 10.3389/fcell.2020.611444

**Published:** 2021-01-05

**Authors:** Lin Cui, Xiuqing Wang, Dekai Zhang

**Affiliations:** ^1^Department of Gastroenterology and Hepatology, The Second Affiliated Hospital, Harbin Medical University, Harbin, China; ^2^Center for Infectious and Inflammatory Diseases, Texas A&M University, Houston, TX, United States

**Keywords:** gastric cancer, toll-like receptor, agonist, tumor immunity, immunotherapy, immune checkpoint

## Abstract

Gastric cancer (GC) is one of the most common cancers in the world, and the incidence of gastric cancer in Asia appears to increase in recent years. Although there is a lot of improvement in treatment approaches, the prognosis of GC is poor. So it is urgent to search for a novel and more effective treatment to improve the survival rate of patients. Both innate immunity and adaptive immunity are important in cancer. In the innate immune system, pattern recognition receptors (PRRs) activate immune responses by recognizing pathogen-associated molecular patterns (PAMPs) and damage-associated molecular patterns (DAMPs). Toll-like receptors (TLRs) are a class of pattern recognition receptors (PRRs). Many studies have reported that TLRs are involved in the occurrence, development, and treatment of GC. Therefore, TLRs are potential targets for immunotherapy to gastric cancer. However, gastric cancer is a heterogeneous disorder, and TLRs function in GC is complex. TLRs agonists can be potentially used not only as therapeutic agents to treat gastric cancer but also as adjuvants in conjunction with other immunotherapies. They might provide a promising new target for GC treatment. In the review, we sort out the mechanism of TLRs involved in tumor immunity and summarize the current progress in TLRs-based therapeutic approaches and other immunotherapies in the treatment of GC.

## Introduction

Gastric cancer (GC), is the second leading cause of cancer death worldwide according to the latest WHO statistics in 2018 (Bray et al., 2018). Early gastric cancer can be removed by EMR (endoscopic mucosal resection) or ESD (endoscopic submucosal dissection), the long-term prognosis is good at present (Ko et al., 2016). What makes GC difficult to treat is due to the patients with GC usually without any early symptoms. GC is usually diagnosed in the late stage when metastasis (Maeda et al., 2015). There are several standard treatments to treat advanced gastric cancer: surgical resection, chemotherapy, radiotherapy, chemoradiation, and targeted therapy. But the 5-year survival rate of patients with GC had no significant improvement. Besides, the patients had some side effects during and after treatment, including nausea, vomiting, reflux, malnutrition, etc. The quality of life of the patients decreased significantly. This makes people with gastric cancer suffer both physically and mentally. How to improve the survival rate and quality of life of GC patients is an urgent problem to be solved. GC is a heterogeneous disorder and some molecular subsets of it exhibit certain characteristics, suggesting that immunotherapy may be an effective treatment (Jones and Smyth, 2020). According to The Cancer Genome Atlas (TCGA) network, GC is classified into the four following molecular subgroups: Epstein Barr virus (EBV), microsatellite instability (MSI), chromosomal instability (CIN) and genomic stable (GS) gastric cancers. In the MSI and EBV subgroups, programmed death-ligand 1 (PD-Ll) is higher expressed, that demonstrate the potential of immune checkpoint inhibitors to treat these GC subtypes (Cancer Genome Atlas Research Network, 2014).

The diseases treated by immunotherapy are through inducing, enhancing, or suppressing immune responses. Compared with currently used drugs, immunomodulatory therapy has fewer adverse side effects and develop less resistance in the treatment of microbial diseases (Masihi, 2001). Active immunotherapy uses the parts of the immune system to enhance tumor immunity (Jeremy et al., 2016). In tumor immunity, the immune system suppresses tumor growth by recognizing and removing tumor cells (Keir et al., 2008). However, tumors can achieve malignant reproduction by immune escape (Hanahan and Weinberg, 2011). Tumorigenesis usually involves inflammatory responses, both innate and acquired immune systems (Akira et al., 2006). It is shown that activating the innate immune system can offset tumor-induced immunosuppression partly and may improve the prognosis of cancer patients (Marcus et al., 2014).

The highly conserved PRRs are a significant component of the innate immune system. PRRs family includes TLRs, nucleotide oligomerization domain (NOD)-like receptors (NLRs), a retinoic acid-inducible gene I (RIG-I) like receptors (RLRs), and C-type lectin receptors (CLRs) (Castaño-Rodríguez et al., 2014). Potential natural immune targets include TLRs, RLRs, and stimulator of interferon genes (STING) (Li et al., 2017). Furthermore, The activation of TLRs is necessary for inducing the adaptive immunity. Therefore, the induction of the innate immune system may work in identifying and antagonizing tumor cells. Although immunotherapy has not become a standard therapy like surgery or chemoradiotherapy, its role in cancer treatment could be significant. Especially TLRs-based strategy has greatly promoted the immunotherapy of lung cancer, melanoma, and renal cell carcinoma, etc., but the function of TLRs in gastric cancer immunotherapy has just attracted more studies. However, there is no previous review on the immunotherapeutic effect of TLRs in gastric cancer. Thus, we summarized the research status and mechanism of TLRs in immunotherapy of gastric cancer.

## TLR Structure and Signaling Pathways

TLRs are type I transmembrane glycoproteins, whose structure includes a leucine-rich repetitive sequence in the extracellular domain, a transmembrane domain, and a conserved Toll/ IL-1R homologous domain (TIR) in the cytosolic region, as they are homologous with the signaling domain of IL-1R family members. Extracellular domain induces homo-dimerization of intracellular TIR by recognition of ligands (except for TLR1/2 and TLR2/6). Then TLRs recruit TIR domain-containing adaptor proteins including myeloid differentiation primary response protein 88 (MyD88) and TIR-domain containing adaptor-inducing interferon-β (TRIF) that initiate signaling pathways to activate the transcription factors nuclear factor-kappa B (NF-κB), interferon regulatory factor (IRFs), or mitogen-activated protein kinase (MAPK) to regulate the expression of cytokine and chemokine genes including interleukin-2 (IL-2), IL-6, IL-12, monocyte chemoattractant protein-1 (MCP-1), and tumor necrosis factor-α (TNF-α) (Barton and Kagan, 2009), ultimately involved in establishing a regulatory innate and adaptive immune response (Goutagny et al., 2012; Kawasaki and Kawai, 2014).

Ten TLRs have been determined expressed in various human innate immune cells, such as macrophages and dendritic cells. TLRs may be retained or abnormally expressed in cancer cells (Goutagny et al., 2012; Braunstein et al., 2018). Several TLRs have been expressed in human gastric cancer cells. For example, TLR2 expression was up-regulated in human GC cell SGC7901, which was associated with Pam3CSK4 stimulation, indicating that TLR2 might be involved in the proliferation and metastasis of GC, indicating that TLR2 might serve as a novel therapeutic target against GC (Yang et al., 2014). TLR4 was highly expressed in gastric cancer cells related to the aggressiveness of gastric cancer. The activation of TLR4 by lipopolysaccharide (LPS) promoted cancer proliferation but did not influence apoptosis (Yuan et al., 2013). TLR5 activated by flagellin can increase the proliferation of GC cells. The subsequent antagonism of TLR5 appears to counteract this effect, suggesting that TLR5 signaling can significantly enhance the proliferation of GC cells (Song et al., 2011). However, another study found that GC patients with high TLR5 tissue expression can have a better prognosis, particularly who has stage II disease or intestinal-type GC (Kasurinen et al., 2019). Studies have explored that TLR7 expression is low in GC tissues, and stimulating TLR7 could promote the secretion of pro-inflammatory cytokines, and inhibit the proliferation of GC cells (Jiang et al., 2016). Thus, it can be seen that the function of different TLRs in GC is complex and different.

## The Mechanism Underlying the Role of TLRs in Tumor Immunity

**Figure 1:**
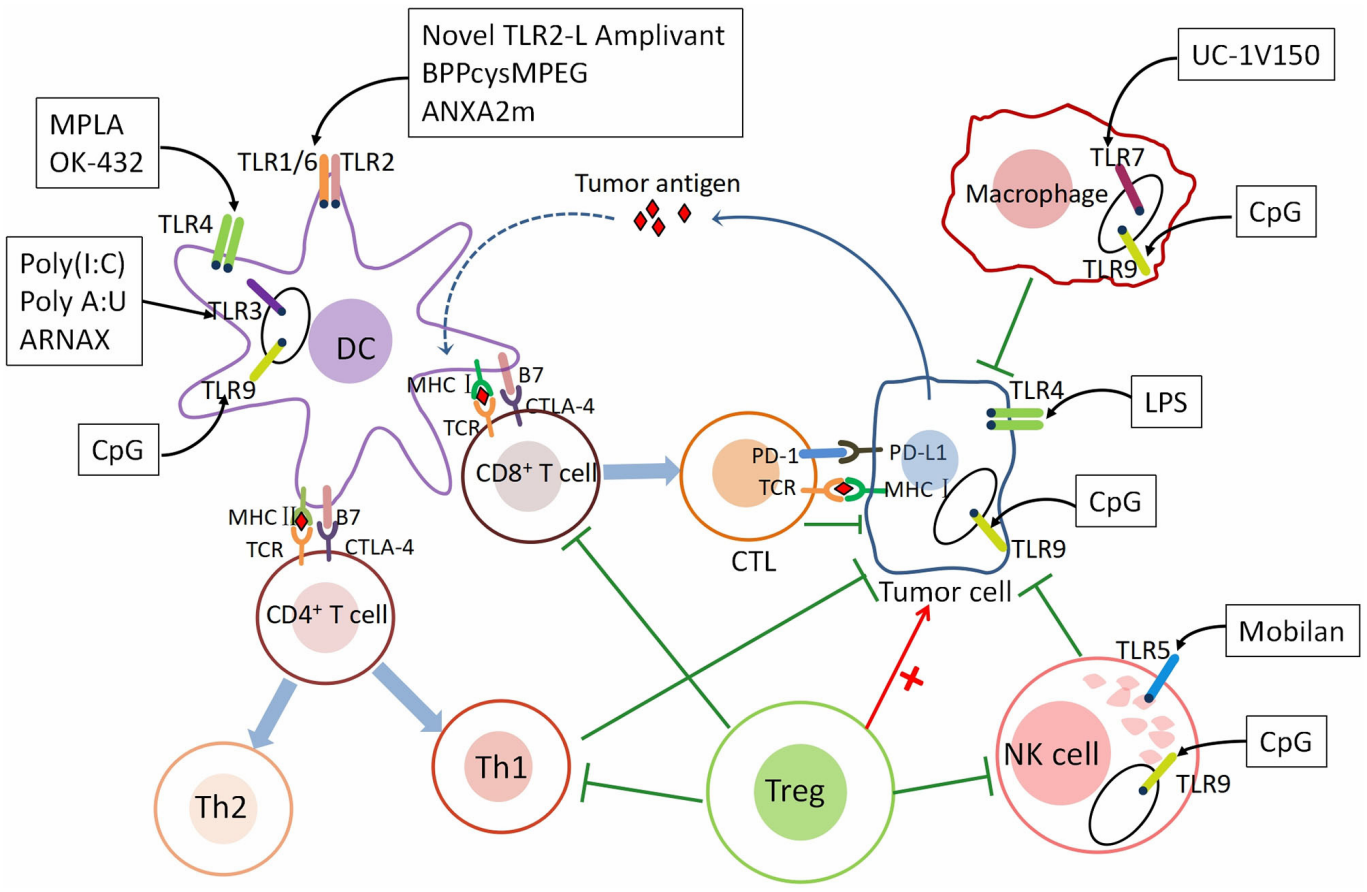
The mechanism underlying the role of TLRs in tumor immunity. TLRs are expressed on different immune cells and cancer cells. The roles of immune cells in tumor immunity. As a class of antigen-presenting cells (APCs), dendritic cells (DCs) recognize tumor antigen and present the antigen to CD8^+^ T cells through the MHC class I pathway and CD4^+^ T cells through the MHC class II pathway. After activated by DCs, CD8^+^ T cells differentiate into cytotoxic T lymphocytes (CTLs) and CD4^+^ T cells differentiate into Th1 and Th2 cells. CTLs are recruited to the tumor microenvironment (TME) and recognize tumor cells through T cell receptor (TCR) and MHC class I-bound antigen, then kill them. Th1 cells secreting proinflammatory cytokines such as IL-2, TNF-α, and IFN-γ to promote immunity. Immune checkpoints—CTLA-4 and PD-1, can suppress T cell response. NK cells inhibit cancer by directly kill tumor cells and secreting several inflammatory factors. M1 macrophages are pro-inflammatory and M2 Macrophages are anti-inflammatory. Regulatory CD4^+^ T cells (Tregs) are anti-inflammatory cells suppressing of CTLs, Th1 cells, macrophages, and NK cells. TLRs are expressed both on immune cells and gastric cancer cells. Different TLRs agonists act on different immune and cancer cells to affect tumor immunity—pro-tumor or anti-tumor. Th1 cells, T helper type 1 cells; Th2 cells, T helper type 2 cells; IL-2, interleukin-2; TNF-α, tumor necrosis factor-α; IFN-γ, interferon-γ; CTLA-4, The cytotoxic T-lymphocyte-associated antigen-4; PD-1, Programmed cell death protein 1; PD-L1, Programmed death ligand 1 (PD-L1).The agonists and adjuvants of TLRs are shown in Tables 1, 2.

TLRs are the family of transmembrane receptors on innate immune cells such as dendritic cells (DCs) and macrophages. They can recognize PAMPs and DAMPs of necrotic and apoptotic cells from a foreign specific microbe, including antigens from various tumor sources, to initiate an immune response (Barton and Kagan, 2009; Tartey and Takeuchi, 2014). The immune system recognizes tumor cells through tumor antigens. Tumor-specific antigen (TSA) is expressed only in tumor cells. Tumor-associated antigen (TAA) is expressed in both tumor and normal cells. Dendritic cells (DCs) belong to antigen-presenting cells (APCs). TAA/TSA was presented to CTLs by DCs via the MHC class I pathway (Kurts et al., 2010). Antigen presentation triggers CD8+ T cells to differentiate into cytotoxic T lymphocytes (CTLs) and induces CTLs proliferation. Subsequently, CTLs are attracted to the tumor microenvironment. Through T cell receptor (TCR) and MHC class I-bound antigen, CTLs recognize tumor cells then kill them (Hanson et al., 2000). Immune checkpoints including CTLA-4 and PD-1 can suppress the immune attack. CD4+ T cells are activated by DCs via the MHC class II pathway, differentiating into Th1 and Th2 cells. Th1 cells promote antitumor immunity by secreting proinflammatory cytokines (Kalams and Walker, 1998). Regulatory CD4+ T cells (Tregs) are anti-inflammatory cells suppressing the priming, activation, and cytotoxicity of other effector immune cells including Th1 T cells, CTLs and etc. (Ward-Hartstonge and Kemp, 2017). TLR agonists who have anti-tumor effects activate tumor-specific T cell responses by stimulating antigen-presenting cells (APC), including dendritic cells (DCs) (Li et al., 2017). T lymphocyte activity, as the main involved molecules in tumor immune response, can synergistically act on stimulating molecules and inhibiting molecules (Pardoll, 2012). In humans, most TLRs expression is found on DCs and mature macrophages (Hennessy et al., 2010). There is growing evidence that TLRs can be expressed or induced on multiple cells, such as T cells and tumor cells. Along with the anti-tumor effect of TLRs on DCs, new researches have shown that TLR signals from other cell types can play a crucial role in tumor growth, promotion or inhibition (Fukata and Abreu, 2008; So and Ouchi, 2010; Kaczanowska et al., 2013). This also indicates that the role of TLRs in immunotherapy against GC is very important, might represent a new strategy for patients with GC (Figure 1).

**Table 1:** The agonists of TLRs in the immunotherapy of gastric cancer.

**TLR**	**TLR agonist/inhibitor**	**Signal**	**Effect**	**Reference**
TLR2	ELL	Activate BMDCs and CTLs	Anti-cancer	Koizumi et al., 2012
TLR2/TLR4	Coley toxin BCG	Induce apotosis and autophagy of gastric cancer cell line MGC-803 (a human gastric cancer cell line)	Anti-cancer	Galluzzi et al., 2012; Yao et al., 2018
TLR3	PolyA:U	Combined with 5-fluorouracil, Adriamycin	Anti-advanced cancer	Jeung et al., 2008
	Poly (I:C)	Overstimulate the immune system	Cause autoimmune and chronic inflammatory diseases	Anders et al., 2005; Lang et al., 2005; Jiang et al., 2008; Zhao et al., 2012; Hafner et al., 2013
TLR4	LPS	promote an suitable environment for the continued proliferation of cancer cells and helping to evade cancer cells from immune surveillance	Pro-cancer	Huang et al., 2005; Tang and Zhu, 2012; Fu et al., 2013; Wang et al., 2013
TLR5	Flagellin	Activate NF-kB	Anti-cancer	Soto et al., 2003; Sfondrini et al., 2006; Rhee et al., 2008; Cai et al., 2011; Burdelya et al., 2012; Garaude et al., 2012
TLR7	Imiquimod	promote the secretion of TNF-α and IL-6, and inhibited cell proliferation in SGC-7901 cells (a human gastric cancer cell line)	Anti-cancer	Jiang et al., 2016
TLR9	Chloroquine(non-specific TLR9 inhibitor)	inhibit the invasion of gastric adenocarcinoma cell line AGS induced by h. pylori DNA.	Anti-cancer	Kauppila et al., 2013

*ELL, the extract of larixleptolepis; Coleytoxin, a mixture of inactivated Streptococcus pyogenes and marcescens Serratia; BCG, Bacillus Calmette-Guerrin; poly A:U, Polyadenylic– polyuridylicacid; Chloroquine is a non-specific TLR9 inhibitor, others are all agonists of TLRs*.

**Table 2:** The adjuvants of TLRs in the immunotherapy of gastric cancer.

**TLR**	**Vaccine**	**Signal**	**Effect**	**Reference**
TLR2	BCG-CWS	Provide immune enhancement through dendritic cells	Anti-gastric cancer	Tsuji et al., 2000
	TLR2-L Amplivant	Induce DCs maturation, T cell priming	Anti-cancer	Zom et al., 2018
	BPPcysMPEG	Generate CTLs via cross-priming	Anti-cancer	Prajeeth et al., 2010
	ANXA2m	Induce DCs maturation	Anti-cancer	Andersen et al., 2016
	lipo-OVA	Activate BMDCs maturation	Anti-cancer	Wu et al., 2016
TLR3	Poly (I:C) -DOTAP liposome complex nanoparticles	enhance the cellular penetration of poly (I:C) and produce corresponding TLR3 signals in BMDCs.	Anti-cancer	Wang et al., 2012
	ARNAX	Targeting TLR3 in DCs, enhancing DC-priming and CTL proliferation	Anti-cancer	Seya et al., 2019
TLR7	T7-MB	Combined with 5-Fu	Anti-gastric cancer	Wang et al., 2018
TLR9	A nanoscale vaccine	Simulates epitope of gastric cancer specific antigen MG7 and adjuvant CpGODN1645	Anti-cancer	Shi et al., 2005

*BCG-CWS, BCG cell wall skeleton; TLR2-L Amplivant, a modified Pam3CSK4, TLR2-ligand; BPPcysMPEG, TLR2/6 heterodimer agonist; ANXA2m, an O2-regulated protein binding and signaling through TLR2; lipo-OVA, ovalbumin fused with a bacterial lipid moiety TLR2 agonist; T7-MB, T7 and MG7-Ag conjugated; T7, TLR7 agonist; MG7-Ag, Monoclonal gastric cancer 7 antigen*.

## TLRs in Immunotherapy of Gastric Cancer

TLRs have been detected in both tumor cells and immune cells. They recognize different ligands, respectively, such as lipopolysaccharide (LPS) of bacteria, bacterial flagellin, unmethylated CpG motif of bacterial, etc. So, the activation of TLRs on different cells is complex, which may be anti-tumor, pro-tumor, or dual effects.

### TLR2

It has been reported in several literatures that TLR2 is associated with the occurrence of GC. TLR2 is expressed on the plasma membrane and recognizes peptidoglycan, a part of the cell wall of Gram-positive bacteria, thus activating innate immunity. It was shown that TLR2-activated APCs can promote effector cells to attack tumor cells (Akazawa et al., 2010; Prajeeth et al., 2010). The extract of LarixLeptolepis (ELL) may activate TLR2 and fight cancer. It can also activate bone marrow-derived-dendritic cells (BMDCs) to promote a tumor-specific signal and tumor-specific cytotoxic T lymphocytes (CTLs) to fight cancer (Koizumi et al., 2012). Coley's toxin (a mixture of inactivated Streptococcus pyogenes and Serratia marcescens) and BCG (Bacillus Calmette-Guerin), a TLR2 and TLR4 agonist have been used as a drug for long-term cancer treatment (Galluzzi et al., 2012). Also, the BCG cell wall skeleton (BCG-CWS) vaccine has been used as a vaccine and TLR2 ligand for the prevention of tuberculosis and has been demonstrated to enhance immune effect strongly through DCs and is helpful for cancer immunotherapy, including GC (Tsuji et al., 2000).

However, the underlying mechanism remains unclear although some information has been uncovered. After BCG and lymphocyte co-culture, the apoptosis rate, caspase-3 level, LC-3, and Atg-3 protein level were significantly higher than those in BCG and lymphocyte group alone. BCG induces lymphocyte to secrete interferon-γ (IFN-γ). BCG and IFN-γ can also enhance the level of cleavage of caspase-3, LC-3, and Atg-5. Studies have shown that BCG may induce apoptosis and autophagy of gastric cancer cell line MGC-803 by inducing the release of IFN-γ from peripheral blood lymphocytes (Yao et al., 2018).

Glycoprotein 130 (gp130) binds to IL-6 to form a trimer and transmit downstream signals (Tebbutt et al., 2002). In gp130 mutant mice, TLR2 was absent and gastric cancer lesion shrank (Tye et al., 2012). And the study indicated that the expression of TLR2 in gastric epithelial cells and/or GC tissues may be involved.

A cancer vaccine is a new approach to treat a tumor, and a vaccine essentially consists of a tumor-associated antigen (TAA) and adjuvant. TLR2 ligands are potential adjuvants. Gijs et al. demonstrated that conjugation of new TLR2-L Amplivant (TLR2-ligand, a modified Pam3CSK4) to synthetic long peptides (SLPs) induced dendritic cells(DCs) maturation, T cell priming, and anti-tumor immunity strongly (Zom et al., 2018). BPPcysMPEG (TLR2/6 heterodimer agonist) was proved to be a potent stimulus to generate cytotoxic T lymphocytes (CTLs) via cross-priming (Prajeeth et al., 2010). Hu et al. designed and synthesized a conjugated stimulator of interferon genes (STING) and TLR1/2 agonist, Pam3CSK4-CDGSF, which can serve as a adjuvant for vaccine construction to augment antitumor immunotherapy (Hu et al., 2020).

ANXA2m is an O2-regulated protein binding and signaling through TLR2, acting as an adjuvant by inducing DCs maturation, enhancing antigen cross-presentation, and inducing the secretion of proinflammatory cytokines (Andersen et al., 2016). Wu et al. found that lipo-OVA (Ovalbumin fused with the TLR2 agonist, the lipid part of the bacteria) showed a strong anti-tumor effect by activating BMDCs maturation, promoting cross-presentation of tumor antigen, inducing CTL responses, increasing the numbers of CD8+ T cells (Wu et al., 2016). A vaccine comprising bacteria-mimicking tumor cells (BMTC) and P2CSR11/P2CSK11 (TLR2 ligand) promoted anti-tumor immunity by stimulating DCs and enhancing antigen presentation (Akazawa et al., 2018).

Above all, TLR2 and its agonists have been proved in many studies to attack gastric cancer cells. The next step is to apply it to clinical trials and verify the efficacy of TLR2-related immunotherapy.

### TLR3

The expression of TLR3 is primarily intracellular. TLR3 was regarded as a potential therapeutic target for multiple cancers. Poly(I:C) which is a classical TLR3 agonist was studied for immunotherapy in recent years. Poly(I:C) was reported that the immune potency of it may be limited by insufficient cellular penetration. Novel immunotherapy was designed based on the cancer vaccine. The Poly(I:C)-DOTAP liposome composite nanoparticles can promote Poly(I:C) into cells and generated corresponding TLR3 signals in BMDCs. In this way, poly(I:C) nanoparticles promoted the tumor properties of TLR3 signals (Wang et al., 2012). The poly(I:C) ligand-receptor complex was internalized into cells by the specific ligand-binding receptors on the surface of tumor cells. TLR3, PKR, RIG-1, and MDA5 was activated by the internalized poly(I:C) simultaneously. The activation of these signaling proteins promotes tumor cells secreting cytokines by the bystander effect and led to the rapid death of tumor cells (Levitzki, 2012). Shime et al. found that TLR3 activated by injection of poly(I:C) to tumor-implant mice converted tumor-supporting macrophages into tumor-killing effectors to shrink the tumor (Shime et al., 2012). Perret et al. demonstrated adding the adjuvant Poly(I:C) to the vaccine increased ratios of tumor antigen-specific effector T cells: regulatory T cells that enhanced the infiltration of CD8+ T-cell, thus promoting anti-tumor immunity. And after treated with OVA+Poly(I:C) vaccine, the tumor growth of EG7 lymphoma was controlled and the survival rate of tumor-bearing mice has enhanced (Perret et al., 2013). Intravaginal (IVAG) instillation of Poly(I:C) after subcutaneous HPV E6/E7 vaccination promoted the proliferation of vaccine-specific CD8+ T cells in the genital mucosa (GM), which may suppress cervical cancer (Domingos-Pereira et al., 2013).

Polyadenylic–polyuridylic acid [poly(A:U)] is also the agonist of TLR3 which can promote immune responses. After surgery for locally advanced gastric cancer, the final results of the 15-year follow-up of phase III clinical trial with 5-fluorouracil and adriamycin vs. 5-fluorouracil, Adriamycin, and poly(A:U) showed that immunochemotherapy had a survival advantage over chemotherapy alone. But this treatment regimen hasn't become standard therapy for GC patients, which may be due to the lack of platinum drugs in the regimen despite its positive effects (Jeung et al., 2008).

However, poly(I:C) has the opposite effect on cancer. The immune system is overstimulated by poly(I:C), leading to autoimmune and chronic inflammatory diseases. Therefore, we strongly recommend the use of poly(I:C) with alow dose and local injection of particle preparation (Anders et al., 2005; Lang et al., 2005; Jiang et al., 2008; Zhao et al., 2012; Hafner et al., 2013). To overcome the effect, a safe adjuvant, called ARNAX, has been developed which targets for TLR3 in dendritic cells (DCs). Compared to poly(I:C), ARNAX hardly induces the production of proinflammatory cytokines, so it does not result in systemic cytokinemia. The combined use of ARNAX/TAA and anti-PD-L1 Ab can induce the resistance of anti-PD-1 (Takeda et al., 2017). It can enhance anti-tumor immunity by promoting DC-priming and CTL proliferation (Seya et al., 2019).

As an adjuvant, TLR3 agonist is a double-edged sword in gastric cancer. However, it still needs a long time to further perfect the research *in vivo* and *in vitro* before it can be used clinically for gastric cancer.

### TLR4

TLR4 is expressed in both tumor and immune cells. The influence of TLR4 on cancer is two-sided, depending on where it is expressed.

Several studies have demonstrated that the expression of TLR4 is increased in various cancer cells and tissues, including gastrointestinal cancers, hepatic cancer, pancreatic cancer, and ovarian cancer (Mai et al., 2013). In gastric cancer, TLR4 is the recognition receptor of helicobacter pylori LPS on gastric epithelial cells (Kawahara et al., 2001; Maeda et al., 2001; Su et al., 2003; Basak et al., 2005). The pro-cancer mechanisms of TLR4 expressing on cancer cells include promoting an environment suitable for the continued proliferation of cancer cells and helping to evade cancer cells from immune surveillance (Huang et al., 2005; Tang and Zhu, 2012; Fu et al., 2013; Wang et al., 2013). For example, LPS-stimulated MC26 (colon cancer) cells supernatants significantly inhibited the function of T cell and NK cell. And in the supernatants, the levels of nitric oxide and IL-6 were higher than controls. So, the production of factors induced by TLR4 signaling is a way to tumor evasion from immune surveillance (Huang et al., 2005). LPS first forms complexes with LPS binding proteins (LBP) and then interacts with monocyte differentiation antigen CD14 and myeloid differentiation protein 2 (MD-2) in turn (Thomas et al., 2002). The complex and TLR4 synergistically induce the MyD88-dependent signaling pathways that lead to transcription factors, which promote inflammation and cancer (Takeda et al., 2003).

Several immune modulators targeting TLR4 have been reported. By binding to and forming a chelate complex with LPS, the TLR4 regulators (antagonists and inhibitors) antagonize the interaction of LPS with CD14 and MD2. TLR4 inhibitors suppress NF-κB signaling, thus reducing inflammation-induced carcinogenesis. For instance, in preclinical models, there is evidence that TLR4 inhibitors can effectively inhibit the development of colon cancer (Kuo et al., 2016) and breast cancer (Yang et al., 2014). It is also suggested as a treatment method for liver cancer (Toffanin et al., 2012). The TLR4 antagonist Ibudilast (AV4II) inhibits the secretion of pro-inflammatory cytokines in neuroinflammation (Ledeboer et al., 2006). This suggests that TLR4 could even be widely used as a primary target for suppressing inflammation-related cancers.

At the same time, activated-TLR4 expressed on immune cells is essential to anti-cancer immunity. Compared with wild-type mice, TLR4-deficient mice grew more tumors after oral tube feeding with carcinogenic polyaromatic aromatic hydrocarbons (PAHs) (Naseemuddin et al., 2012). TLR4 agonists induce maturation of dendritic cells (DCs), promoting the immune response of cancer-antigen specific cytotoxic T cells (Fang et al., 2014), which ultimately kill cancer cells. Mainly based on the mechanism, TLR4 agonists have immunomodulatory effects as adjuvants in vaccines, chronic viral infection therapy, and cancer therapy. Jang et al. identified 60S acidic ribosomal protein P2 (RPLP2) by pull-down assay using human cancer derived proteins that binds to TLR4. Recombinant RPLP2 induced maturation and activation of DCs *in vitro*. This DC-based vaccine has been shown to improve both tumor prevention and tumor treatment *in vivo* (Jang et al., 2020). Monophosphoryllipids A (MPLA) with low toxicity, a modified lipopolysaccharide derivative, retains most of the immune-stimulating activity. It is an immunomodulatory agent that stimulates T cell priming by activating the TRIF-associated TLR4 signaling pathway, not MyD88 (Casella and Mitchell, 2008). MPLA has been approved as part of the hepatitis B and human papillomavirus vaccines (Krieg, 2007), but whether it can have the same effect in gastric cancer remains to be seen. OK-432(Picibanil), an anticancer agent, was reported acting at least partly via TLR4-MD2 signaling pathway and inducing maturation of DCs (Ryoma et al., 2004). In a case report, a squamous cell carcinoma patient was treated with intratumoral injection of OK-432, then the tumor size diminished and no metastasis occurred during a follow-up of 5 years (Akeda et al., 2012). Studies have shown that OK-432 adjuvant immunochemotherapy may have marginal and significant efficacy in stage III or IV patients after radical gastrectomy of gastric cancer (Oba et al., 2016).

As the most classical TLR in gastric cancer caused by helicobacter pylori, TLR4 has great value in immunotherapy of gastric cancer. From the current background, TLR4 stimulation is a double-edged sword, so more researches should be done on gastric cancer patients, and then search for the balance of anti-tumor and pro-tumor in the future.

### TLR5

Many studies have suggested that TLR5 is an effective target for antitumor immunotherapy. Bacterial flagellin, a TLR5 agonist, has been demonstrated strong anti-cancer effect in lots of animal models (Soto et al., 2003; Sfondrini et al., 2006; Rhee et al., 2008; Cai et al., 2011; Burdelya et al., 2012; Garaude et al., 2012). The anti-cancer effects of TLR5 agonists stem from the dependence on TLR5 to activate NF-κB. Flagellin is a potent catalyst for NF-κB signaling that mediates natural and acquired anti-tumor immune responses. Nucleoside diphosphate kinase 3(NME3) can enhance flagellin signaling. The expression of NME3 is highly associated with the expression of TLR5 in GC. High NME3 expression reduces the overall survival rate of gastric cancer. In summary, NME3 may strengthen cancer immunotherapy as an unrecognized pro-inflammatory cytokine in TLR5 downstream signaling (Flentie et al., 2018). An adenovirus-based tumor-specific delivery vector, called Mobilan, drives the expression of the TLR5 signaling cassette, which is composed of salmonella flagellin and humanTLR5, which is similar in structure to the clinical-stage TLR5 agonist entolimod. The injection of Mobilan was injected into the primary tumors of transgenic adenocarcinoma mice susceptible to prostate cancer (TRAMP), resulting in a powerful induction of a couple of genes involved in inflammation and mobilization of natural immune cells, to suppress tumor progression (Mett et al., 2018). The high expression of TLR5 in the tissues may determine the better prognosis of patients with GC, especially those with intestinal-type and stage II advanced GC (Kasurinen et al., 2019). Conversely, TLR5 agonist was reported that can induce an inflammatory response and inhibited some tumor progression. Therefore, the mechanism of TLR5 in immunotherapy of GC deserves further study.

### TLR7

TLR7 is expressed on the endosomal membrane (Takeda and Akira, 2015). TLR7 was found low expressed in GC tissues (Jiang et al., 2016). Activating TLR7 can strengthen anti-cancer immunity. Ma et.al developed a bi-functional vector containing a Bcl-2-silencing shRNA and a TLR7-stimulating ssRNA, which promoted apoptosis and inhibited cell growth in MFC cells (a mouse gastric cancer cell line) (Minaga et al., 2017). UC-1V150 which is a TLR7 agonist binds to phospholipids to increase levels of pro-inflammatory cytokines (Wu et al., 2007). A typical TLR7 agonist-imiquimod who has a non-nucleoside imidazolquinoline structure, can be used in immunotherapy of superficial basal cell carcinoma (Papadavid et al., 2007). Imiquimod can induce the expression of TLR7 protein, promote inflammatory cytokines secreting, and inhibited cell proliferation in one of the human gastric cancer cell lines (Jiang et al., 2016). It is also being studied as an adjuvant to anti-tumor vaccines. However, imiquimod lacks the chemical groups that are associated with proteins or peptides. Another TLR7 agonist (T7) has a free carboxyl group that can be attached to amino peptides (Chan et al., 2009). Small molecule T7 was covalently attached to gastric cancer antigen to construct a series of vaccines. After being introduced into animal models, the vaccines could generate immunogenic stimulation and tumor inhibition (Wang et al., 2015). Tumor-associated antigen (TAA) is a target of anti-cancer immunotherapy (Zhang et al., 2006). The monoclonal gastric cancer 7 antigen (MG7-Ag), a TAA in GC, has higher specificity and selectivity compared with existing antigens. T7 and MG7-Ag were conjugated together to construct a new gastric cancer vaccine T7–MB. The inhibition effect of 5-Fu and T7–MB on tumor size and volume by treatment regimen was higher than that of 5-Fu or T7–MB alone (Wang et al., 2018). Overall, either used alone or used as an adjuvant, TLR7 agonist has been shown as an anti-tumor agent in immunotherapy of gastric cancer currently.

### TLR9

TLR9 is expressed in many cancer tissues and cell lines, including gastric, hepatocellular, prostate, and colorectal cancers (Damiano et al., 2007; Brignole et al., 2010; So and Ouchi, 2010). TLR9 can play the role of both anti-tumor and pro-tumor. For example, in hepatocellular carcinoma cell lines, the activation of TLR-9 inhibits apoptosis by IL-8, IL-1, IL-6 up-regulation, thereby increasing proliferation and inflammation. In neuroblastoma cells, the activation of TLR-9 results in increased cystatin-dependent apoptotic cell death (Brignole et al., 2010).

TLR-9 can enhance anti-tumor immunity by recognizing Oligodeoxynucleotide(ODN) containing cytosine–guanine dinucleotide (CpG), which is a potential therapeutic agent. Immunosuppressive cells characteristically exist in the immune microenvironment, one of which is Myeloid suppressor cells (MDSC). A large number of MDSCs cluster near the tumor and inhibit the activity of T cells and NK cells. Several reports showed that the agonist CpG ODN of TLR9 can kill tumors by reducing the immunosuppressive activity of monocyte MDSC11-12. At the same time, CpG ODN can be used as a powerful adjuvant to many antigens. CpG ODN promotes the Th1 response (Weiner, 2000; Mccluskie et al., 2002), and when used in combination with the immunogen, enhances immune responses (Yamamoto et al., 2000). It directly activates monocytes/macrophages, DCs, etc., and inducts pro-inflammatory cytokines (Wagner et al., 2000; Gramzinski et al., 2001; Liu et al., 2003). Especially when CpG ODN is tightly bound to the antigen, the activity will be improved. Xu et al. first conducted polyethyleneimine-CpG nanocomplex (CpG@PEI) as an *in situ* vaccine for melanoma *in vivo* therapy (Xu et al., 2020). Shi et al. constructed a nanoscale vaccine that simulates epitope of gastric cancer-specific antigen MG7 and adjuvant CpGODN1645, may have important significance for tumor treatment (Shi et al., 2005).

Kauppila et al. found that Helicobacter pylori DNA can promote the invasion of gastric adenocarcinoma cell line AGS, while chloroquine (non-specific TLR9 inhibitor) can inhibit the invasion induced by Helicobacter pylori DNA (Kauppila et al., 2013). Qin et al. found that in human gastric cancer cells, H. pylori DNA can up-regulate TLR9 expression, promoting cell proliferation, migration, and invasion. In a word, TLR9 is involved in the occurrence and development of gastric cancer (Qin et al., 2019).

A TLR9 agonist can be used as an adjuvant in immunotherapy to make the checkpoint inhibitors more effective. Fumi et al. confirmed that intratumoral administration of 1V270 (TLR7 agonist) or SD-101(TLR9 agonist) and blocking PD-1 can suppress the development of primary tumors and prevent tumor metastasis in the head and neck squamous cell carcinoma (HNSCC) model. This treatment is more effective than any drug alone (Sato-Kaneko et al., 2017). Reilley et al. demonstrated that intratumoral administration of ODN1826 (TLR9 agonist) can synergistically block ctLA-4 and promote the rejection of bilateral implantation of B16 ovalbumin (B16-OVA) melanoma (Reilley et al., 2019).

Above all, we can see that no matter whether the function of TLR9 is pro- or anti-cancer, it is important in the immunotherapy of cancer. How to use its function to treat gastric cancer deserves further study.

## Current Immunotherapies for Gastric Cancer

According to TCGA network classification, gastric cancer is divided into four molecular subtypes: Epstein-Barr virus (EBV), microsatellite instability (MSI), genomically stable (GS), and chromosomal instability (CIN)gastric cancers (Sohn et al., 2017). PD-L1 is more likely expressed on the EBV and MSI subtype (Ma et al., 2016).

At present, trastuzumab targeting HER2 has become the standard therapeutics for advanced gastric cancer as a molecular targeted therapy (Abrahao-Machado and Scapulatempo-Neto, 2016), but the prognosis of patients is still poor. Therefore, immunotherapy for gastric cancer has emerged recently.

### Immune Checkpoint Inhibitors

The immune system eliminating cancer cells depends on the cancer-immune cycle, including recognition, stimulation, recruitment, amplification, and final memory to inhibit cell growth. T lymphocytes work together to stimulate and inhibit molecules, which act as immune checkpoints. Through immune checkpoint pathways, T cell suppression signals are released allowing tumors to evade immune surveillance and destruction, thus achieving malignant proliferation (Hanahan and Weinberg, 2011; Pardoll, 2012). Programmed death receptor-1 (PD-1) is mainly expressed on the surface of activated T cells and B cells, and is the receptor involved in cell death. Programmed death receptor-ligand 1 (PD-L1) is binding to PD-1, expressed in antigen-presenting cells(APC), such as macrophages, DCs, and cancer cells. Prolonged TCR stimulation induces the secretion of interferon-γ (IFN-γ) to increased PD-L1 in cancer cells (Buchbinder and Desai, 2016). PD-L1 binding to PD-1 decreases the function and cytotoxicity of effector T cell. The interaction of CD28 on T cells with B7-1/2 on APCs is essential for T cell activation. Cytotoxic T lymphocyte-associated antigen 4 (CTLA-4) has a much higher binding affinity with B7, but not promoting T cell activation (Leach et al., 1996). So the molecule has a non-overlapping inhibitory effect against tumor immunity and participates in the early stage of an immune response of lymphoid organs (Abrahao-Machado and Scapulatempo-Neto, 2016). When immune checkpoint inhibitors are combined with PD-1, PD-L1, and CTLA-4, the function of T lymphocytes can't be suppressed, and the immune response is activated against cancer (Coutzac et al., 2019).

An anti-PD-1 antibody binds to PD-1 to prevent the combination of PD-1 and PD-L1. Blocking the PD-1 pathway inhibits the negative immune regulation of the PD-1 pathway on T cells, then the anti-tumor response is stimulated (Ribas et al., 2015; Nghiem et al., 2016; Bellmunt et al., 2017; Eggermont et al., 2018; Fuchs et al., 2018; Gandhi et al., 2018; Zhu et al., 2018; Chung et al., 2019). Pembrolizumab is a high-affinity anti-PD-1 humanized monoclonal antibody targeting the PD-1 receptor and is already approved for treating GC. A phase 1 study of patients with advanced PD-1 positive GC has shown that pembrolizumab had good antitumor activity and controlled toxicity, which needs further verification in phase 2 and 3 trials (Muro et al., 2016).

Nivolumab, a humanized monoclonal immunoglobulin G4 antibody, is also a PD-1 receptor blocking antibody. It has been approved as a treatment for advanced gastric cancer in Japan. In a phase III randomized study, the ONO-4538-12 (ATTRACTION-2) trial, patients receiving chemotherapy for unresectable late-stage gastric esophageal cancer were divided into the nivolumab 3 mg/kg group and the placebo group (Kang et al., 2017). Nivolumab improved the median overall survival rate. Of patients treated with nivolumab, the recurrence rate was 12% and the tumor reduction rate was 40%. Nivolumab can improve the survival of patients regardless of whether there is PD-L1 expression (Boku et al., 2017). Therefore, regardless of the state of PD-L1, nivolumab is effective for chemotherapeutic gastric cancer.

Anti-PD-L1 antibodies reverse the anti-inflammatory effect of PD-1/PD-L1 pathway and contribute to the anticancer response by binding to PD-L1. Comparative study of Avelumab (anti-PD-L1 antibody) and chemotherapy in the treatment of gastric cancer/gastroesophageal junction cancer showed that the clinical activity of avelumab is not superior to chemotherapy, but avelumab is safer than chemotherapy (Bang et al., 2018).

Durvalumab (the anti–PD-L1 antibody) and tremelimumab (the anti–CTLA-4 antibody) can enhance T cells response to the tumor by blocking the immune checkpoint together, thus generating anti-tumor activity. A Phase Ib/II study in patients with advanced GC showed durvalumab and tremelimumab have a manageable safety profile in the second-line and third-line treatment, with encouraging survival rate compared to durvalumab alone (Kelly et al., 2020).

Ipilimumab is a monoclonal antibody binding to CTLA-4, which can enhance T cells activation and promote tumor immunity. A report compared ipilimumab monotherapy with best supportive care in GC patients who have stabilized during first-line chemotherapy. Compared with the latter, the progression-free survival of the former wasn't improved. In ipilimumab group, the median overall survival rate is ~1 year and ipilimumab has good safety. So, the drugs combined with other therapies for advanced GC is worth for futherstudies (Bang et al., 2017).

It can be seen that not all patients can benefit from immune checkpoint inhibitor therapy alone. As many patients with GC are CIN and GS, the immune signal expresses lowly, and the response to immunotherapy may be weak, but the safety is an advantage compared with traditional radiotherapy and chemotherapy. The combination of immunotherapy inhibitors has been used more and more to improve the response of immunotherapy and has achieved remarkable results.

### Checkpoint Blockade Immunotherapy

Checkpoint blocking immunotherapy revolutionizes cancer treatment and improves patient survival and quality of life. However, the effects of this immunotherapy are highly heterogeneous between patients (Chuang et al., 2020). Response rates of patients to checkpoint blockade range from 15 to 30% in most solid tumors (Das and Johnson, 2019). It is urgent to find effective adjuvant treatments to improve the efficacy of immune checkpoint inhibitors. Tumors with low numbers of tumor-infiltrating T cells tend to respond poorly to immune checkpoint blockade therapy (Tsukamoto et al., 2019). As mentioned earlier, TLRs play an important role in immune response. So TLRs agonists may be potential adjuvants for treating overcome checkpoint blockade resistance, to increase efficacy of checkpoint blockade therapy.

It has been shown that the infiltration of CD8+ T cells in tumor site is essential to anti-tumor effect of PD-1 blockade therapy (Tumeh et al., 2014). Many studies have confirmed that TLRs agonists can increase the level of CD8+ T cells or activate CD8+ T cells.

Pam3CSK4, a TLR1/2 ligand, increased Fcγ receptor IV expression on macrophages. The macrophage mediated depletion of Tregs. Through the mechanism, Pam3CSK4 can increase efficacy of anti-CTLA-4 antibody (Chuang et al., 2020). A unique structural domain (UNE-C1) was identified as a novel TLR2/6 ligand that can activate dendritic cells to produce a powerful humoral and cellular immune response *in vivo*. UNE-C1 also showed synergistic effects *in vivo* with tumor antigens and checkpoint inhibitors in different cancer models (Cho et al., 2020). ARNAX, a TLR3 agonist, promoted full priming and proliferation of CTLs and enhanced CD8+ T cell infifiltration into the tumor site through TLR3-TIR domain-containing adaptor molecule-1 (TICAM-1)-IRF3-IFN-β axis in DCs. Therapy using ARNAX/TAA can overcome anti-PD-L1 resistance (Takeda et al., 2017). OVA/TLR4 mAb can enhance the anti-tumor effect of anti-PD-1 mAb by activating OVA-specific CD8+ T-cells (Tsukamoto et al., 2019). CIRP is a TLR4 ligand. SIIN-CIRP up-regulated the expression of PD-L1 on DC, but at the same time enhanced T cell response. It was finally confirmed that the combination of the SIIN-CIRP vaccine with antibodies against PD-1 can inhibit tumor growth (Villanueva et al., 2018).

Combining anti-PD-1 with CMP-001 (a TLR-9 agonist) to treat head and neck squamous cell carcinoma (HNSCC) had a better effect and prolonged the survival time of mouse, compared with anti-PD-1 alone. And CMP-001+anti-PD-1 induced anti-tumor response depends on activation of CD8+ T cells (Cheng et al., 2020). In addition to depending on CD8+ cells, there are other mechanisms that promote the efficacy of checkpoint blockade therapy. CpG is a TLR9 ligand. It can down-regulate PD-1 expresssion on CD8+ T cells by inducing the production of IL-12 from dendritic cells (Yin et al., 2016). Thomas et al. confirmed the combination of CpG with CTLA-4 or PD-1 blockade not only increased the levels of circulating tumor-specific CD8+ T cells, but also reduced the number of Tregs at the tumor site (Mangsbo et al., 2010). Lefitolimod, also a TLR9 agonist, can induce the secretion of IFN-α by qDC. IFN-α increased the number of CD8+ T cells and anti-tumoral M1 macrophages, activated NK-cells and decreased pro-tumoral M2 macrophages inside the TME. In colon carcinoma CT26 model, the combined treatment with lefitolimod and anti-PD-L1 inhibited tumor growth and prolonged survival of the mice (Kapp et al., 2019). Radiation can directly induce upregulation of PD-L1 on tumor cells, and also act as an adaptive resistance mechanism for T cells to release IFNγ in tumor microenvironment. There is a hypothesis that the addition of targeted immunosuppressive PD-1/PD-L1 checkpoint to the radiation /TLR regimen may enhance the antitumor immune response (Walshaw et al., 2020).

**Figure 2:**
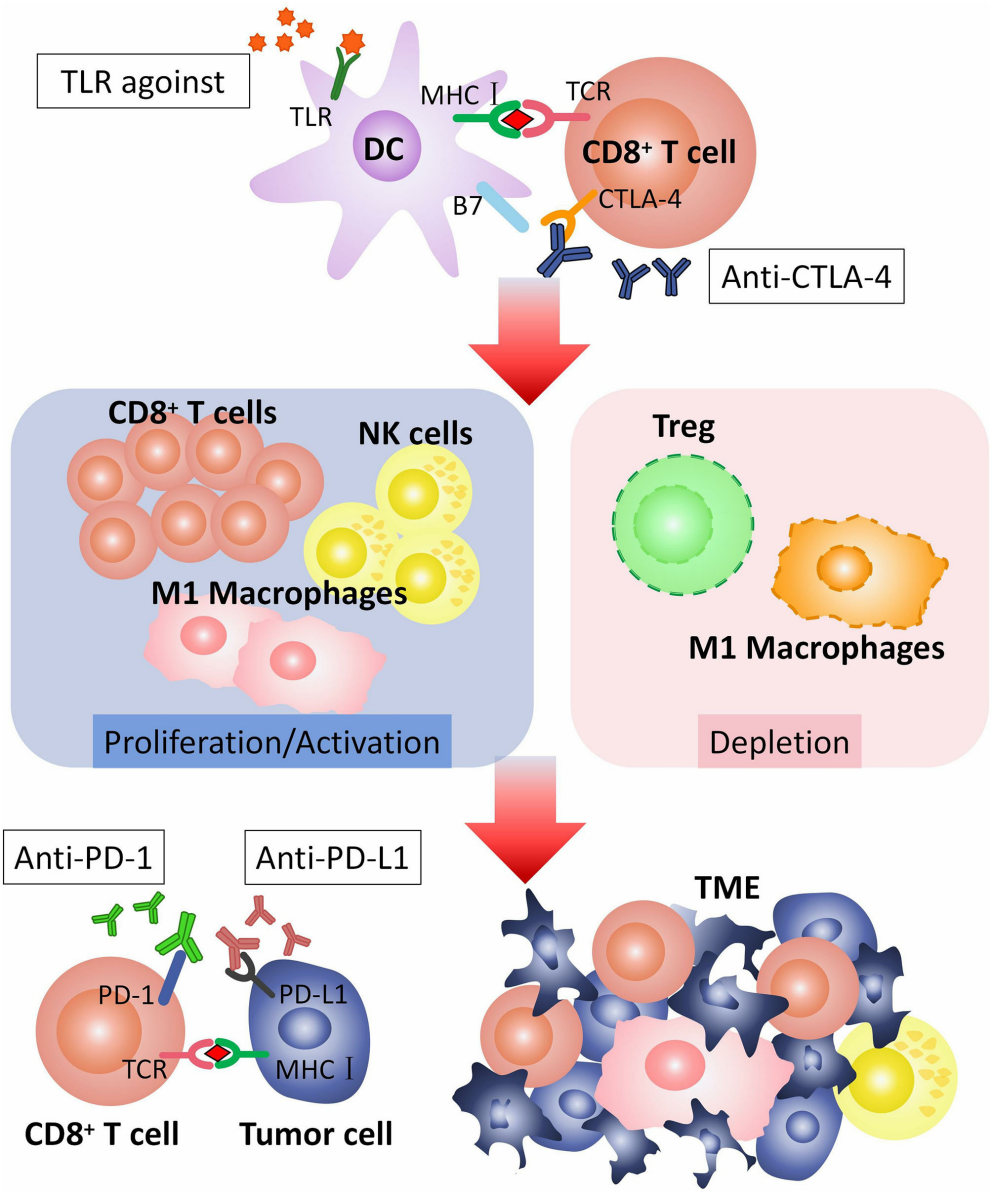
How TLR agonist promotes the efficacy of checkpoint blockade therapy. CTLA-4 is expressed on the surface of CD8^+^ T cell, combining with B7 on dendritic cells (DCs), to regulate T cell responses. CTLA-4 pathway can be blocked by anti-CTLA-4 antibodies. TLR agonist activates TLR on DC. Activated TLRs pathway can induce proliferation or activation of CD8^+^ T cells, M1 macrophages and NK-cells, and depletion of M2 macrophages and Tregs. CD8^+^ T cells, M1 macrophages and NK-cells recognize and kill tumor cells in TME. PD-1 is expressed on CD8^+^ T cell. PD-L1 is expressed on tumor cell. The combination of PD-1 with PD-L1 inhibits the anti-tumor effect of T cells, that can be blocked by anti-PD-1 or anti-PD-L1 antibodies. TLR agonist and anti-CTLA-4/anti-PD-1/anti-PD-L1 antibodies play a synergistic role in anti-tumor immunotherapy. TLR, toll-like receptor; TCR, T cell receptor; CTLA-4, The cytotoxic T-lymphocyte-associated antigen-4; PD-1, Programmed cell death protein 1; PD-L1, Programmed death ligand 1; TME, Tumor microenvironment.

In conclusion, TLRs agonists can promote efficacy of checkpoint blockade therapy by inducing or activating CD8+ T cells, M1 macrophages and NK-cells, decreasing M2 macrophages and Tregs, and down-regulating PD-1 expresssion on CD8+ T cells. TLRs agonists are potential adjuvants for checkpoint blockade therapy (Figure 2).

### Tumor Antigen Vaccine

Tumor-associated antigen (TAAs) can activate the immune response. By generating an immune response, cancer vaccines transform tumor-specific T cells into effector T cells. Therapeutic vaccines can enhance autoimmunity, leading to a stronger anti-tumor immune response. In order to induce tumor-specific T cells, peptides produced by TAAs must be provided to T cells by antigen-specific presenting cells to play an immune role and destroy tumor cells. The peptides derived from TAAs are presented to the T cells by APCs to play an immune role and destroy tumor cells (Steinman, 1991). Specific immune response in GC can be activated by protein and peptide targets. These studies are based on TAA peptide extracted from HER2/neu and MAGE. TAA peptide induces cytotoxic T cells to fight cancers, restricted to MHC class I (Tanaka et al., 1997; Kono et al., 1998). Typical HER2/neu overexpression in gastric cancer and dendritic cells immunizing HER2/neu peptide pulses can lead to tumor regression. The MAGE-3 peptide/chitosan-deoxycholic acid inoculation nanoparticles have been successfully used to stimulate an anti-tumor immune response and inhibit the growth of tumors in mice with GC (Yang et al., 2010). The potential of combining tumor antigen vaccines with conventional chemotherapy to strengthen the anti-cancer response has been the focus of current research.

TLR agonists have been reported that they can contribute to anti-tumor immunity as adjuvants in conjunction with other immunotherapies. It seems that combination therapy is more effective in immunotherapy.

## Conclusion

The immunotherapy of gastric cancer is a hot topic in recent years. TLRs are expressed not only in natural immune cells but also in non-immune cells, like tumor cells. TLRs play a crucial role in innate immunity. Many studies show that TLRs have complex functions in tumor immunity including pro-tumor and anti-tumor. There is considerable evidence that TLRs play an important role in the occurrence and development of GC, can recognize the composition of Helicobacter pylori, a major cause for GC. TLRs are increasingly being used as immunotherapeutic targets for cancer and infectious diseases. TLR ligands show their anti-tumor activity not only as therapeutic agents directly but also as adjuvants in conjunction with tumor-associated antigen (TAA) or immune checkpoint inhibitors. TLRs agonists are potential adjuvants for checkpoint blockade therapy. Although TLR-based immunotherapy is imperfect and has not been widely used in the clinic yet, elucidating the mechanism of TLR signaling in anti-tumor response and its interaction with other signaling pathways is meaningful. TLRs may provide a promising novel strategy for gastric cancer treatment.

## Author Contributions

LC performed literature searches, and drafted the manuscript. XW drew figures and supplemented the manuscript. DZ revised the manuscript. LC and DZ developed the idea for this article. All authors have approved the final version of this submission.

## Conflict of Interest

The authors declare that the research was conducted in the absence of any commercial or financial relationships that could be construed as a potential conflict of interest.
